# Moral sensitivity as a personal and work attribute of emergency care nurses: a cross-sectional study*

**DOI:** 10.1590/1518-8345.7178.4311

**Published:** 2024-09-02

**Authors:** Mariana Oliveira Antunes Ferraz, Carlise Rigon Dalla Nora, Maria Manuela Ferreira Pereira da Silva Martins, Rosinete Souza Barata, Larissa Dantas Ferreira, Darci de Oliveira Santa Rosa

**Affiliations:** 1 Universidade Estadual do Sudoeste da Bahia, Jequié, BA, Brazil.; 2 Universidade Federal do Rio Grande do Sul, Escola de Enfermagem, Porto Alegre, RS, Brazil.; 3 Universidade do Porto Instituto de Ciências Biomédicas Abel Salazar, Ciências Biomédicas, Porto, PO, Portugal.; 4 Universidade Estadual de Montes Claros, Enfermagem, Montes Claros, MG, Brazil.; 5 Universidade Federal da Bahia, Escola de Enfermagem, Salvador, BA, Brazil.

**Keywords:** Nursing Care, Emergencies, Emergency Nursing, Nurses, Ethics Nursing, Morals

## Abstract

**Objective:**

to analyze the moral sensitivity of Brazilian emergency care nurses according to their personal and work characteristics.

**Method:**

this is a quantitative, descriptive, cross-sectional study with a convenience sample. A total of 422 nurses from emergency care services in the five regions of Brazil took part. Sociodemographic and work-related information was collected, as well as the Brazilian version of the Moral Sensitivity Questionnaire. After approval by the Research Ethics Committee, the data was collected using a self-administered form on the Google Forms Platform, organized using Excel software and analyzed using the R language.

**Results:**

nurses with longer experience in emergency care services showed higher levels in the interpersonal orientation, moral conflict and moral significance dimensions, while in the professional knowledge dimension, men showed higher levels, as evidenced by items that include confidence in nursing knowledge, intuition, experience and opinion.

**Conclusion:**

the differences in the nurses’ moral sensitivity were due to their professional experience. It should be emphasized that valuing the sharing of intergenerational experiences in service could be a possible strategy for fostering moral competencies in the field of practice.

## Highlights

**(1)** Nurses’ understanding of moral sensitivity (MS).

**(2)**Moral sensitivity of emergency care nurses.

**(3)** Valuing the sharing of intergenerational experiences.

**(4)** Strategy for fostering moral competencies.

**(5)** Distinctions in moral sensitivity related to professional experience.

## Introduction

There are situations that can interfere with nurses providing safe and qualified care in the context of emergency care services, such as the work processes and interpersonal relationships established in this care setting^1^. These situations may or may not reveal possible conflicts experienced by nurses with regard to their duties, responsibilities and values when providing health care. Thus, making decisions in the face of perceived moral conflicts, understood here as any event in which there is contradiction, opposition or confrontation over principles, values and attitudes^2^, requires a series of skills from these professionals.

Among the skills evoked to recognize ethically sensitive situations and resolve conflicts, moral competence is indispensable to health care practice. This is because the quality of care is related to both the clinical and moral skills of nurses^3^.

The component of moral competence involved in the beginning of the reflective process and the recognition of morally sensitive situations is moral sensitivity (MS), which is relevant for professionals to be able to deal with the complexities of care, especially in contexts of greater vulnerability^4^. This sensitivity roles as an individual awakening to the moral issues involved in a context, which leads to sources of conflict being perceived and dealt with. Thus, recognizing the conflicts arising from the practice of care becomes a key element that triggers the decision-making process and without this, the quality of nursing care can be compromised^5^.

Since moral sensitivity is a complex phenomenon present in professional training and practice contexts, and various elements interact in its development, it is important to delve deeper into the phenomenon,paying attention to the different scenarios in which nursing operates^4^.

Professional practice favors the development of moral sensitivity, since it is in this context that the real dimension of ethical problems is perceived^6^. Thus, nurses’ experience has been associated with the development of moral sensitivity^7^ ^)-(^ ^8^. Some studies have tried to establish relationships between individual and work characteristics, such as gender and workplace, and moral sensitivity, with different conclusions about these associations^7^ ^),(^ ^9^ ^)-(^ ^10^. 

In a literature review on the interaction between moral sensitivity and care, three studies addressed the moral sensitivity of nurses in primary health care and hospital units in Brazil^4^. There are no studies that delve into moral sensitivity in the context of Brazilian emergency departments. However, international studies have been carried out with nursing professionals in the context of emergency services, such as in Iran, where an empowerment program was promoted for nurses and in the evaluation, the impact was greater moral sensitivity in the intervention group^11^.

In the quest to deepen our knowledge of the phenomenon in the context of emergency services, given the specificities of care in this area, this study aims to analyze the moral sensitivity of nurses working in Brazilian emergency services according to their personal and work characteristics.

## Method

### Type of study

This is a quantitative, cross-sectional study whose report was guided by the Strengthening the Reporting of Observational Studies in Epidemiology (STROBE) verification items for observational studies.

### Period

Data was collected from February to June 2022.

### Population

According to a national nursing survey, there are 85,773 nurses working in Urgent and Emergency Hospital Care, UPA and SAMU throughout Brazil^12^. The study participants were nurses working in Brazilian emergency services.

### Selection criteria

The criterion for including participants was working as a nurse in emergency care services, and those among the eligible were excluded if they refused to agree virtually to the Informed Consent Form (ICF), and if they were not working at the time of the data collection, which was presented in an exclusion question in the process of filling in the instrument. 

### Participants

The sample was non-probabilistic, for convenience, since the study took place during the pandemic period, when access to potential participants was restricted. 

A total of 422 nurses working in Brazilian emergency services took part in the study, based on the spontaneity of the professionals in accessing and answering the instrument. There were 751 accesses to the instrument during the data collection period, 11 people did not agree with the term or did not agree to take part in the study, 319 were not working in emergency services at the time of completion, which resulted in the participants in this study.

### Instruments used to collect information

The Moral Sensitivity Questionnaire (MSQ-B), translated and validated in Brazil(13), was used to assess nurses’ moral sensitivity. For this study, 19 items were considered which had previously been submitted to a study to assess evidence of the validity of the internal structure, in which it showed a good internal consistency index, assessed using McDonald’s omega ωMSQ-B-19= 0.812. 

The items were distributed in the following dimensions: interpersonal orientation, the motivation for social contact, perceived in nursing as attention to relationships in search of trust; moral meaning, refers to giving meaning to actions taken based on reflection and structuring meaning; experiencing moral conflict, refers to the perception of conflict; and confidence in knowledge, portrays the conviction that knowledge is necessary to deal with moral issues^13^ ^)-(^ ^14^. The answers were obtained on a Likert scale from 1 (totally disagree) to 7 (totally agree). 

### Data collection

Data was collected online using a self-administered form on the Google Forms Platform containing the Brazilian version of the Moral Sensitivity Questionnaire, which was disseminated on social networks to reach all five Brazilian regions. 

The researchers’ network of contacts, the Regional Nursing Councils and Postgraduate Nursing Programs, contacted by the researcher in charge, who requested support on social networks, via email and/or Instagram, contributed to the dissemination of the online instrument. 

### Data processing and analysis

The data was tabulated in Excel software and analyzed using the R programming language (R CORE TEAM). A Permutation Multivariate Analysis of Variance (PERMANOVA) was carried out as the data was asymmetrical. The assumption of sample normality was investigated using the Kolmogorov-Smirnov test and the p-value was considered significant at 0.05.

The multivariate model was then tested using the factors from the moral sensitivity questionnaire as dependent variables: a) Interpersonal Orientation; b) Professional Knowledge; c) Moral Conflict; d) Moral Meaning. 

The independent variables (i.e. group delimiters) were: I) Sex (female and male); II) Position (hospital emergency, Mobile Emergency Care Service 192, Emergency Care Units and other emergency services); III) Role (direct assistance, coordination, regulation and other activity considered by the participant); IV) Hours of work (up to 30 hours, 31 to 44 hours and over 44 hours); and V) Length of experience (up to 5 years, 6 to 15 years and over 16 years).

### Ethical aspects

The study was approved by the Research Ethics Committee of the Nursing School of the Federal University of Bahia, under opinion no. 5.141.402 and Certificate of Submission for Ethical Appraisal (CAAE) 53607021.2.0000.5531. The participants were informed about the objectives, benefits and risks of the research and the researcher’s contact details for any further questions or clarifications were included in the Informed Consent Form, a copy of which was sent to the e-mail address registered by each participant.

## Results

Of the participants, 322 (78.7%) were female, which is why we chose to use the term nurse(s) to describe them in the study. Most of the sample worked in the hospital emergency department at the time of data collection (n= 145, 34.4%), with most of the participants working in direct patient care (n= 329, 78%), as well as having up to 5 years’ experience in the emergency department (n= 181, 42.9%), and working between 30 and 44 hours (n= 253, 60%). The other characteristics of the participants and the tests carried out are presented below (Table 1).

**Table 1:** Profile of nurses working in emergency services according to personal and professional characteristics (n = 422). Salvador, BA, Brazil, 2022

Variables		n*	%
**Sex**	Female	332	78.67%
	Male	90	21.33%
**Position**	Hospital emergency	145	34.36%
	Other urgent and emergency services	92	21.80%
	SAMU^†^ 192	62	14.69%
	24-hour UPA^‡^/Emergency medical services	123	29.15%
**Role**	Direct patient care	329	77.96%
	Coordination	70	16.59%
	Other activities	18	4.26%
	Regulation	5	1.18%
**Working hours**	Up to 30 hours	106	25.12%
	31 to 44 hours	253	59.95%
	Over 44 hours	63	14.93%
**Length of professional expertise**	Up to 5 years	181	42.89%
	From 6 to 15 years	177	41.94%
	16 years or more	64	15.17%

*n = Absolute Frequency; ^†^SAMU = Mobile Emergency Care Service; ^‡^UPA = Emergency Care Unit

Initially, the normality tests were investigated using the Kolmogorov-Smirnov test, which indicated that the distribution of the dependent variables was asymmetrical in all cases. In this way, the inferential tests that follow are configured as non-parametric alternatives for the investigation. 

The multivariate model was then carried out considering 9,999 resamples. The PERMANOVA results indicated statistically significant differences only for the Length of experience [F(2)= 3.540, p = 0.032, R2= 0.016], as well as the interactions between Hours of Work and Time of Experience [F(2)= 3.126, p = 0.045, R2= 0.014], and between Sex, Role and Hours of Work [F(1)= 10.518, p = 0.002, R2= 0.024] (Table 2).

**Table 2:** Multivariate model of moral sensitivity (n = 422). Salvador, BA, Brazil, 2022

	F*	df^†^	*p* ^*‡*^	R^2§^
Sex	2.471	1	0.115	0.005
Position	1.168	3	0.318	0.008
Role	0.221	3	0.882	0.001
Working hours	1.285	1	0.260	0.003
Length of experience	3.540	2	0.032^||^	0.016
Hours of work/Time of experience	3.126	2	0.045^||^	0.014
Sex/Role/Working hours	10.518	1	0.002^||^	0.024

*F = PERMANOVA Test; ^†^df = Degrees of Freedom; ^‡^p = Significance Level; ^§^R2 = Variance Explained; ^||^Statistically Significant

Table 3 shows the results of the univariate models of the instrument’s factors/dimensions. 

**Table 3:** Univariate models by moral sensitivity dimension (n = 422). Salvador, BA, Brazil, 2022

Variables	F*	df^†^	*p* ^*‡*^	R^2§^
**Interpersonal Orientation Dimension**				
Sex	0.883	1	0.352	0.002
Position	0.645	3	0.593	0.004
Role	0.612	3	0.613	0.004
Working hours	2.869	1	0.089	0.006
Length of experience	4.711	2	0.008^||^	0.021
Sex/Role	3.817	3	0.010^||^	0.026
Service/Time of experience	2.332	6	0.033^||^	0.032
Sex/Role/Hours of work	7.927	1	0.006^||^	0.018
**Professional Knowledge Dimension**				
Sex	4.381	1	0.034^||^	0.009
Position	0.943	3	0.415	0.006
Role	0.775	3	0.520	0.005
Working hours	0.177	1	0.672	0.000
Sex/Role	3.207	3	0.022^||^	0.021
Service/Time of experience	2.104	6	0.049^||^	0.028
Hours of work/Time of experience	8.028	2	< 0.001^||^	0.035
**Moral conflict dimension**				
Sex	0.939	1	0.331	0.002
Position	1.433	3	0.230	0.010
Role	0.189	3	0.903	0.001
Working hours	0.182	1	0.669	0.000
Length of experience	4.350	2	0.013^||^	0.020
Sex/Role/Hours of work	13.567	2	< 0.001^||^	0.032
**Moral meaning dimension**				
Sex	0.243	1	0.622	0.000
Position	2.340	3	0.076	0.015
Role	1.160	3	0.329	0.007
Working hours	0.331	1	0.557	0.000
Length of experience	4.749	2	0.008^||^	0.021
Sex/Service/Role/Time of experience	3.190	2	0.041^||^	0.014

*F = PERMANOVA Test; ^†^df = Degrees of Freedom; ^‡^p = Significance Level; ^§^R2 = Variance Explained; ^||^Statistically Significant

Considering only the Interpersonal Orientation variable, there were significant differences between the variables: Length of Experience [F(2)= 4.711, p = 0.008, R2= 0.021], as well as the interactions between Sex and Position [F(3)= 3.817, p = 0.010, R2= 0.026], Position and Length of Experience [F(6)= 2.332, p = 0.033, R2= 0.032], and Sex, Position and Working Hours [F(1)= 7.927, p = 0.006, R2= 0.018].

In addition, the univariate model for the Professional Knowledge variable showed significant differences for Sex [F(1)= 4.381, p = 0.034, R2= 0.009], as well as the interactions between Sex and Position [F(3)= 3, 207, p = 0.022, R2= 0.021], Position and Length of Experience [F(6)= 2.104, p = 0.049, p = 0.028], and Hours of Work with Length of Experience [F(2)= 8.028, p < 0.001, R2= 0.035]. 

Subsequently, the univariate model considering Moral Conflict showed significant differences for the Time of Experience groups [F(2)= 4.350, p = 0.013, R2= 0.020], as well as the interaction between Sex, Position and Working Hours [F(2)= 13.567, p < 0.001, R2= 0.032].

Finally, the univariate model considering the Moral Meaning variable showed statistically significant differences between the Time of Experience groups [F(2)= 4.749, p = 0.008, R2= 0.021], as well as the interaction between Sex, Position, Role and Time of Experience [F(2)= 3.190, p = 0.041, R2= 0.014]. 

Paired comparisons were then made between the groups. Table 4 shows the paired comparisons by dimension which showed significant differences between the groups.

**Table 4:** Paired comparisons of the groups that maintained significant statistical differences according to the univariate model (n = 422). Salvador, BA, Brazil, 2022

Interpersonal orientation dimension					
	Pairs	F*	R^2†^	*p* ^*‡*^	I-J^§^
Length of experience	Up to 5 years - 16 years and over	6.586	0.026	0.036^||^	0.364
	6 to 15 years - 16 years and over	8.619	0.035	0.021^||^	0.400
**Professional knowledge dimension**					
	**Pairs**	F*	**R** ^2†^	***p*** ^*‡*^	**I-J** ^§^
Sex	Female-Male	4.117	0.010	0.048^||^	-0.308
**Moral conflict dimension**					
	**Pairs**	F*	**R** ^2†^	***p*** ^*‡*^	**I-J** ^§^
Length of experience	16 years and over - up to 5 years	7.719	0.030	0.027^||^	0.522
	16 years and over - 5 to 16 years	7.695	0.031	0.024^||^	0.493
**Moral meaning dimension**					
	**Pairs**	F*	**R** ^2†^	***p*** ^*‡*^	**I-J** ^§^
Length of experience	16 years and over - Up to 5 years	8.017	0.032	0.024^||^	0.507

*F = PERMANOVA Test; ^†^R2 = Variance Explained; ^‡^p = Significance Level; ^§^I-J = Difference between the mean response between the groups; ^||^Statistically Significant

The results show that people with 16 years or more experience had higher levels of Interpersonal Orientation when compared to people with up to 5 years’ experience (I-J= 0.364, p = 0.021) and between 6 and 15 years (I-J= 0.400, p = 0.021).

The paired comparisons indicated that women had lower levels of Professional Knowledge when compared to men (I-J= -0.308, p = 0.048). 

Professionals with 16 or more years’ experience had higher levels of Moral Conflict when compared to professionals with up to 5 years’ experience (I-J= 0.522, p = 0.027), and professionals with between 5 and 16 years’ experience (I-J= 0.493, p = 0.024). 

Nurses with 16 years’ experience or more had higher levels of Moral Meaning when compared to people with up to 5 years’ experience (I-J= 0.507, p = 0.024). 

In the other variables presented in the univariate model by dimension, it was not possible to observe any other paired comparison, leading us to believe that the overall effects found for the interactions were not confirmed when comparing the groups pair by pair.

## Discussion

From the analysis of the moral sensitivity of nurses working in emergency services in Brazil, some highlights were given to their personal and work characteristics. To begin with, when describing the participants, it can be seen that the distribution of the sample by gender was mostly female, similar to other contexts in which MS has been studied in nursing professionals, such as in Intensive Care Units (ICUs) in Turkey^7^, in end-of-life care in South Korea^15^ and among Brazilian nurses^16^. 

This proportion differs from another study in the context of emergency care in Turkey, where a more homogeneous sample was presented between the sexes, which revealed 52.5% of women and 47.5% of men^17^. In another study conducted in medical clinics in Iran, the proportion of women reached 96.5%^18^. This may indicate that, although the profile of professionals has changed over the years, nursing is still a profession with a significant participation of women, due to the history and social issues related to the constitution of the profession.

With regard to the existence of differences of opinion on the influence of a professional’s gender on their MS, evaluating this characteristic in isolation can lead to misinterpretations. A study showing differences in moral sensitivity according to the sex of the professionals found no significant differences between men and women^7^. However, the data presented in this study indicate a significant difference in relation to the professional knowledge dimension, which portrays confidence in nursing knowledge and the appreciation of intuitive knowledge and experience by professionals to help them make difficult decisions, with men showing higher rates.

When it comes to professional knowledge, a study of ICU nurses in the southern region of Brazil showed that the search for knowledge promotes the ability to question the facts that occur and recognize inappropriate situations in the work context in which they are inserted^6^, thus being an important dimension in the development of the moral sensitivity of these professionals.

It should be pointed out that MS does not develop in the same way in all aspects of life^19^, i.e. a person may be more sensitive in certain contexts than in others, which increases the importance of experience for the development of moral sensitivity in the context of professional work. Professional experience in this study was considered in terms of the time spent working in the emergency service. 

Cognitivism believes that moral principles are the result of innate or acquired knowledge, whether intuitive or demonstrative^2^. Thus, the quality of experiences and cognition (knowledge and skills learned) develop perceptions of how actions influence others, and memories and judgments are involved in this process. 

The ethical climate interferes with the development of the individual’s moral competences, so environments where ethical issues are not valued, problems are not identified and resolved, which can damage moral sensitivity, leading to a phenomenon that is recognized as moral neutralization^3^.

This is relevant because one of the characteristics of this sensitivity is the use of professional experiences as a tool to recognize the ethical aspects involved in a given situation. Therefore, in the best conditions for moral development, the longer the time in practice, the greater the nurses’ moral competence tends to be to use skills developed over time in decision-making^20^. 

Furthermore, the findings of this study corroborate the length of experience in emergency care as the professional characteristic that stood out in the distinctions between the groups in the dimensions of MH. Thus, when it comes to the Interpersonal Orientation dimension, these findings suggest that people with 16 years’ experience or more have higher levels of this orientation when compared to people with up to 5 years’ experience. 

The interpersonal orientation dimension was the one that showed the greatest agreement between nurses working in different contexts, including studies with culturally distinct populations^4^. This dimension corresponds to the behaviors developed by professionals in search of a relationship of trust with the patient and alternatives to meet their needs^13^, as there is a professional concern about how their actions affect their relationship with the person under care^14^. In a study with Primary Health Care (PHC) health staff, the interpersonal relationship was identified by the participants as promoting new perceptions, which favor the development of MS^4^. 

When relationships of trust are not established between nurses and the people they care for, they reinforce the emergence of ethical problems^21^. Strengthening these relationships is not limited to know-how, a more technical aspect of care, but integrates dimensions of the human sphere, which are interconnected with respect, from the nurses’ recognition of the uniqueness of the person under care and demonstration that in order to practice the profession, respect for the human condition is fundamental, as well as multi-professional relationships that must be established in order to provide the best care^5^. 

Thus, when nurses seek to know the patient as a whole, they are better able to establish a respectful relationship as perceived in emergency services^21^. A study on the relationship between moral sensitivity and the ethical climate at work found a positive association between this sensitivity and maintaining the privacy of the person being cared for^22^. They also come to recognize the needs of the people under their care, which are sometimes veiled by the dynamics of the service, so moral sensitivity provides them with the skills to recognize and deal with conflicts^5^.

In terms of the Moral Conflict dimension, nurses with more experience are those who perceive conflict the most. MH is a marker of moral competence, as it is involved in both recognizing and improving conflict resolution skills^5^. It is through moral awareness that nurses recruit their knowledge of professional ethics and become aware of possible conflicts in their work environment^20^. Thus, when experience is reflected upon, it provides a basis for resolving similar conflicts in the future. 

However, nurses sometimes encounter situations in which their professional values diverge from institutional values, or they find it difficult to deal with other colleagues and work professionals when they are faced with a patient^23^. 

These situations reinforce the need to understand the moral significance of practice, which should not be structured in isolation according to external expectations of care, especially when these diverge from the greater good, which is the production of care. After all, the care that nursing proposes as a means of achieving the goal of the well-being of others is based on principles that guarantee respect for the person being cared for, even within the confines of the institution^24^, for example.

In the nurses’ moral significance dimension, there was also evidence of a relationship between this dimension and length of experience, which refers to giving meaning to the actions taken to meet the needs of the person being cared for^14^. Thus, it can be inferred that contexts in which the autonomy of the person is reduced, as can be seen in emergency services^25^, can lead nurses to feel more morally responsible for the care provided. As a result, there seems to be greater agreement on the moral significance of situations in which they experience conflicts, such as those in which they make decisions without the person’s participation.

The limitations of this study arise from not having a probabilistic sample, which restricts the generalization of the data. Also, due to the need to obtain evaluations in order to establish cut-off points for the instrument; and the participants’ low adherence, which may be related to restricted access to internet networks in some regions.

One of the study’s contributions is that it can provide a better understanding of the mental health of nurses working in emergency services in Brazil, by expanding the subject in this field. This understanding is relevant because it points to the need for meaningful practices and experiences to be promoted right from professional training, contributing to the development of MS in future nurses and, as a result, strengthening their decisions based on ethical, patient-centered care.

## Conclusion

When analyzing the moral sensitivity of emergency nurses, integrating personal and work data in the sample studied, it was possible to identify that length of experience in the service had a higher impact on the phenomenon under study. In addition, males had higher rates of confidence in knowledge, which invites us to look more deeply into the issues of gender and professional confidence. 

It should be noted that valuing the sharing of intergenerational experiences in service with professionals could be a possible strategy for fostering moral competences in the field of practice, while at the same time valuing the body of knowledge built up over time. However, due to the study’s methodological limitations, particularly the type of sampling, we suggest parsimony when it comes to generalizing the data.
