# Supplementary material for: Moral sensitivity as a personal and work attribute of emergency care nurses: a cross-sectional study
Source: Rev Lat Am Enfermagem. 2024 Sep 2;32:e4311. doi: 10.1590/1518-8345.7178.4311 (PMC11386486; doi:10.1590/1518-8345.7178.4311)
Supplement: Translation

# Translation: Sensibilidade moral como atributo pessoal e de trabalho das enfermeiras nas urgências: estudo transversal*

**DOI:** 10.1590/1518-8345.7178.4312

**Authors:** Mariana Oliveira Antunes Ferraz, Carlise Rigon Dalla Nora, Maria Manuela Ferreira Pereira da Silva Martins, Rosinete Souza Barata, Larissa Dantas Ferreira, Darci de Oliveira Santa Rosa

**Affiliations:** 1 Universidade Estadual do Sudoeste da Bahia, Jequié, BA, Brasil.; 2 Universidade Federal do Rio Grande do Sul, Escola de Enfermagem, Porto Alegre, RS, Brasil.; 3 Universidade do Porto Instituto de Ciências Biomédicas Abel Salazar, Ciências Biomédicas, Porto, PO, Portugal.; 4 Universidade Estadual de Montes Claros, Enfermagem, Montes Claros, MG, Brasil.; 5 Universidade Federal da Bahia, Escola de Enfermagem, Salvador, BA, Brasil.

**Keywords:** Cuidado de Enfermagem, Emergências, Enfermagem em Emergência, Enfermeiras e Enfermeiros, Ética em Enfermagem, Princípios Morais

## Abstract

**Objetivo:**

analisar a sensibilidade moral das enfermeiras que atuam em serviços brasileiros de urgência conforme suas características pessoais e de trabalho.

**Método:**

pesquisa quantitativa, descritiva, transversal e com amostra por conveniência. Participaram 422 enfermeiras dos serviços de atenção à urgência das cinco regiões do território brasileiro. Foram coletadas informações sociodemográficas e laborais, e a versão brasileira do *Moral Sensitivity Questionnaire*. Os dados foram coletados, após aprovação no Comitê de Ética, por meio de um formulário autoaplicado na Plataforma *Google Forms*, sendo submetidos à organização pelo *software Excel* e analisados via linguagem R.

**Resultados:**

as enfermeiras com maior tempo de experiências nos serviços de atenção às urgências apresentaram maiores níveis nas dimensões de orientação interpessoal, conflito moral e significado moral, já na dimensão conhecimento profissional, os homens apresentaram maiores níveis, evidenciado por itens que incluem a confiança no conhecimento de enfermagem, na intuição, experiência e opinião.

**Conclusão:**

as distinções da sensibilidade moral das enfermeiras se deu quanto à experiência profissional. Destaca-se que valorizar o compartilhamento de vivências e experiências intergeracionais em serviço, pode ser uma possível estratégia para fomentar competências morais no campo de prática.

## Destaques

**(1)** Compreensão sobre a sensibilidade moral (SM) de enfermeiras.

**(2)** Sensibilidade moral das enfermeiras da urgência.

**(3)** Valorizar o compartilhamento de vivências e experiências intergeracionais.

**(4)** Estratégia para fomentar competências morais.

**(5)** Distinções da sensibilidade moral relacionada à experiência profissional.

## Introdução

Há situações que podem interferir no desempenho do cuidado seguro e qualificado das enfermeiras no contexto dos serviços de atenção às urgências, como, por exemplo, os processos de trabalho e as relações interpessoais instituídas no cenário de cuidado^1^. Tais situações podem deixar aparentes, ou não, possíveis conflitos vivenciados pelas enfermeiras, sobretudo no que concerne aos seus deveres, responsabilidades e valores durante a prestação de cuidados em saúde. Desse modo, tomar decisões diante da percepção dos conflitos morais - aqui entendidos como todo acontecimento no qual se viva uma contradição, oposição ou enfrentamento acerca de princípios, valores, atitudes^2^ - requer dessas profissionais uma série de competências.

Dentre as competências evocadas para reconhecer situações sensíveis do ponto de vista ético e resolver os conflitos, a competência moral torna-se indispensável à prática assistencial em saúde. Isso porque a qualidade do cuidado está tanto relacionada às habilidades clínicas quanto às morais das enfermeiras^3^. 

O componente da competência moral envolvido, no início do processo reflexivo e de reconhecimento das situações moralmente sensíveis, é a sensibilidade moral (SM), sendo um componente relevante para que o profissional possa lidar frente às complexidades do cuidado, especialmente em contextos de maiores vulnerabilidades^4^. 

A sensibilidade funciona como um despertar individual das questões morais envolvidas em um contexto, o que faz com que as fontes de conflitos sejam percebidas e enfrentadas. Assim, reconhecer os conflitos advindos da prática do cuidado torna-se um elemento-chave disparador do processo de tomada de decisão e sem isso pode haver comprometimento na qualidade da assistência de enfermagem^5^.

Considerando que a sensibilidade moral é um fenômeno complexo presente em contextos de formação e de prática profissional, e que diversos elementos interagem no seu desenvolvimento, fundamenta-se o aprofundamento do fenômeno com atenção aos distintos cenários de atuação da enfermagem^4^. 

O exercício profissional favorece o desenvolvimento da sensibilidade moral, pois é nesse contexto que a real dimensão dos problemas éticos é percebida^6^. Assim, a experiência das enfermeiras tem sido associada ao desenvolvimento da sensibilidade moral^7^ ^)-(^ ^8^. Alguns estudos buscaram estabelecer relações entre características individuais e de trabalho com a sensibilidade moral, como o sexo e o local de trabalho, obtendo-se distintas conclusões acerca dessas associações^7^ ^),(^ ^9^ ^)-(^ ^10^.

Em revisão da literatura sobre a interação entre a sensibilidade moral e o cuidado, três estudos abordaram a SM das enfermeiras na atenção primária à saúde em unidades hospitalares no Brasil^4^. Não há estudos que aprofundem a sensibilidade moral no contexto das urgências brasileiras. Entretanto, estudos internacionais foram realizados com os profissionais de enfermagem que atendem nos serviços de urgências, como no Irã, em que foi promovido um programa de empoderamento para os enfermeiros. Na avaliação, o impacto foi a maior sensibilidade moral no grupo da intervenção^11^.

Na busca por aprofundar os conhecimentos em torno do fenômeno no contexto das urgências, dada as especificidades do cuidado nessa área, este estudo tem como objetivo analisar a sensibilidade moral das enfermeiras que atuam em serviços brasileiros de urgência conforme suas características pessoais e de trabalho.

## Método

### Tipo do estudo

Trata-se de pesquisa quantitativa e transversal, cujo relatório foi norteado pelos itens de verificação *Strengthening the Reporting of Observational Studies in Epidemiology* (STROBE) para estudos observacionais.

### Período

A coleta dos dados ocorreu no período de fevereiro a junho de 2022.

### População

Em pesquisa nacional da enfermagem, existem 85.773 enfermeiras e enfermeiros atuantes na Assistência Hospitalar de Urgência e Emergência, UPA e SAMU, em todo o Brasil^12^. As participantes do estudo foram enfermeiras atuantes em serviços brasileiros de urgência.

### Critérios de seleção

O critério para inclusão de participante foi: atuar como enfermeira em serviços de atenção à urgência. Foram excluídas aquelas, dentre as elegíveis, que se recusassem a registrar concordância, virtualmente, do Termo de Consentimento Livre e Esclarecido (TCLE), além daquelas que não exerciam a função no momento da coleta, apresentada em uma questão de exclusão do processo de preenchimento do instrumento.

### Participantes

A amostra foi não probabilística por conveniência devido ao período de realização do estudo, que ocorreu ainda na pandemia, em que o acesso aos potenciais participantes era restrito. 

Participaram do estudo enfermeiras atuantes em serviços brasileiros de urgência, captadas a partir da espontaneidade das profissionais em acessar e responder o instrumento, o qual possuiu 751 acessos, no período da coleta de dados. Desse número, 11 pessoas não concordaram com o termo ou não aceitaram participar do estudo e 319 não trabalhavam em serviços de urgência no momento do preenchimento, o que resultou em 422 participantes para o estudo.

### Instrumentos utilizados para a coleta das informações

Para avaliar a sensibilidade moral dos enfermeiras foi inserido o Questionário de Sensibilidade Moral (MSQ-B), traduzido e validado no Brasil^13^. Para este estudo, foram considerados 19 itens previamente submetidos ao estudo de avaliação da evidência de validade de estrutura interna, que apresentou bons índices de consistência interna, avaliada a partir do ômega de McDonald ωMSQ-B-19= 0,812. 

Os itens estavam distribuídos nas dimensões: orientação interpessoal - isto é, a motivação do contato social, percebido na enfermagem como a atenção no relacionamento em busca da confiança; significado moral - refere-se a dar sentido às ações tomadas a partir da reflexão e estruturação do sentido; vivência do conflito moral - diz respeito à percepção sobre o conflito; e confiança no conhecimento - retrata a convicção de que o conhecimento é necessário para lidar com as questões morais^13^ ^)-(^ ^14^. As respostas eram obtidas em escala do tipo Likert de 1 (discordo totalmente) a 7 (concordo totalmente). 

### Coleta de dados

A coleta de dados foi conduzida no formato *online*, a partir de formulário autoaplicado na Plataforma *Google Forms*, contendo a versão brasileira do *Moral Sensitivity Questionnaire*, cuja divulgação ocorreu em redes sociais, para o alcance das cinco regiões brasileiras. 

Contribuíram para a divulgação do instrumento *online* a rede de contatos das pesquisadoras, os Conselhos Regionais de Enfermagem e os Programas de Pós-graduação em Enfermagem, contatados pela pesquisadora responsável, a qual solicitou o apoio nas redes sociais, ocorrendo por *e-mail* e/ou *Instagram*. 

### Tratamento e análise dos dados

Os dados foram tabulados no *software Excel* e analisados via linguagem R de programação (R CORE TEAM). Realizou-se uma Análise Multivariada de Variância por Permutações (PERMANOVA), por se tratarem de dados assimétricos. O pressuposto da normalidade da amostra foi investigado através do teste de Kolmogorov-Smirnov e os valores considerados significativos quanto ao p-valor foi de 0,05.

A partir disso, o modelo multivariado foi testado utilizando como variáveis dependentes os fatores do questionário da sensibilidade moral, sendo eles: a) Orientação Interpessoal; b) Conhecimento Profissional; c) Conflito Moral; d) Significado Moral. 

As variáveis independentes (i.e., delimitadoras de grupos), foram: I) Sexo (feminino e masculino); II) Serviço (emergência hospitalar, Serviço de Atendimento Móvel de Urgência 192, Unidades de Pronto Atendimento e outros serviços de urgência); III) Função (assistência direta, coordenação, regulação e outra atividade considerada pelo participante); IV) Horas de trabalho (até 30 horas, 31 a 44 horas e acima de 44 horas); e V) Tempo de experiência (até 5 anos, 6 a 15 anos e acima de 16 anos).

### Aspectos éticos

O estudo obteve aprovação pelo Comitê de Ética em Pesquisa da Escola de Enfermagem da Universidade Federal da Bahia, sob parecer nº 5.141.402 e Certificado de Apresentação de Apreciação Ética (CAAE) 53607021.2.0000.5531. As participantes foram informadas e esclarecidas sobre os objetivos, benefícios e riscos da pesquisa e os contatos das pesquisadoras para dúvidas e esclarecimentos adicionais presente no TCLE, o qual teve uma cópia encaminhada para o *e-mail* cadastrado por cada participante.

## Resultados

Das participantes, 322 (78,7%) foram do sexo feminino, por isso optou-se em reportar, no estudo, o termo enfermeira(s) para a sua descrição. A maior parte da amostra trabalhava no serviço de emergência hospitalar no momento da coleta de dados (n= 145, 34,4%), a função de assistência direta ao paciente apresentou a maior parte das participantes (n= 329, 78%), além do tempo de experiência no serviço de urgência de até 5 anos (n= 181, 42,9%), e as horas de trabalho na faixa acima de 30 até 44 horas (n= 253, 60%). A seguir apresentam-se as demais características das participantes bem como os testes realizados (Tabela 1).

**Tabela 1:** Perfil das enfermeiras atuantes nos serviços de urgência conforme características pessoais e profissionais (n = 422). Salvador, BA, Brasil, 2022

Variáveis		n*	%
**Sexo**	Feminino	332	78,67%
	Masculino	90	21,33%
**Serviço**	Emergência hospitalar	145	34,36%
	Outro serviço de Urgência e Emergência	92	21,80%
	SAMU^†^ 192	62	14,69%
	UPA^‡^ 24 horas / Pronto atendimento	123	29,15%
**Função**	Assistência direta ao paciente	329	77,96%
	Coordenação	70	16,59%
	Outra atividade	18	4,26%
	Regulação	5	1,18%
**Horas de trabalho**	Até 30 horas	106	25,12%
	De 31 a 44 horas	253	59,95%
	Acima de 44 horas	63	14,93%
**Tempo de experiência**	Até 5 anos	181	42,89%
	De 6 a 15 anos	177	41,94%
	16 anos ou mais	64	15,17%

*n = Frequência Absoluta; ^†^SAMU = Serviço de Atendimento Móvel de Urgência; ^‡^UPA = Unidade de Pronto Atendimento

Inicialmente, os testes de normalidade foram investigados por meio do teste de Kolmogorov-Smirnov, que apontaram para a assimetria da distribuição das variáveis dependentes em todos os casos. Dessa maneira, os testes inferenciais que se seguem são configurados em alternativas não paramétricas para a investigação. 

A partir disso, o modelo multivariado foi realizado considerando 9.999 reamostragens. Os resultados da PERMANOVA indicaram diferenças estatisticamente significativas somente para as variáveis Tempo de Experiência [F(2)= 3,540, *p* = 0,032, R^2^= 0,016], além das interações entre Horas de Trabalho e Tempo de Experiência [F(2)= 3,126, *p* = 0,045, R^2^= 0,014], e entre Sexo, Função e Horas de Trabalho [F(1)= 10,518, *p* = 0,002, R^2^= 0,024] (Tabela 2). 

**Tabela 2:** Modelo multivariado da sensibilidade moral (n = 422). Salvador, BA, Brasil, 2022

	F*	df^†^	*p* ^*‡*^	R^2§^
Sexo	2,471	1	0,115	0,005
Serviço	1,168	3	0,318	0,008
Função	0,221	3	0,882	0,001
Horas de trabalho	1,285	1	0,260	0,003
Tempo de experiência	3,540	2	0,032^||^	0,016
Horas de trabalho/tempo de experiência	3,126	2	0,045^||^	0,014
Sexo/Função/Horas de trabalho	10,518	1	0,002^||^	0,024

*F = Teste PERMANOVA; ^†^df = Graus de Liberdade; ^‡^p = Nível de Significância; ^§^R^2^ = Variância Explicada; ^||^Estatisticamente significativo

A Tabela 3 apresenta resultados sobre os modelos univariados dos fatores/dimensões do instrumento. 

**Tabela 3:** Modelos univariados por dimensão da sensibilidade moral (n = 422). Salvador, BA, Brasil, 2022

Variáveis	F*	df^†^	*p* ^*‡*^	R^2§^
**Dimensão Orientação Interpessoal**				
Sexo	0,883	1	0,352	0,002
Serviço	0,645	3	0,593	0,004
Função	0,612	3	0,613	0,004
Horas de trabalho	2,869	1	0,089	0,006
Tempo de experiência	4,711	2	0,008^||^	0,021
Sexo/Função	3,817	3	0,010^||^	0,026
Serviço/Tempo de experiência	2,332	6	0,033^||^	0,032
Sexo/Função/Horas de trabalho	7,927	1	0,006^||^	0,018
**Dimensão Conhecimento Profissional**				
Sexo	4,381	1	0,034^||^	0,009
Serviço	0,943	3	0,415	0,006
Função	0,775	3	0,520	0,005
Horas de trabalho	0,177	1	0,672	0,000
Sexo/Função	3,207	3	0,022^||^	0,021
Serviço/Tempo de experiência	2,104	6	0,049^||^	0,028
Horas de trabalho/Tempo de experiência	8,028	2	< 0,001^||^	0,035
**Dimensão Conflito Moral**				
Sexo	0,939	1	0,331	0,002
Serviço	1,433	3	0,230	0,010
Função	0,189	3	0,903	0,001
Horas de trabalho	0,182	1	0,669	0,000
Tempo de experiência	4,350	2	0,013^||^	0,020
Sexo/Função/Horas de trabalho	13,567	2	< 0,001^||^	0,032
**Dimensão Significado Moral**				
Sexo	0,243	1	0,622	0,000
Serviço	2,340	3	0,076	0,015
Função	1,160	3	0,329	0,007
Horas de trabalho	0,331	1	0,557	0,000
Tempo de experiência	4,749	2	0,008^||^	0,021
Sexo/Serviço/Função/Tempo de experiência	3,190	2	0,041^||^	0,014

*F = Teste PERMANOVA; ^†^df = Graus de Liberdade; ^‡^p = Nível de Significância; ^§^R^2^ = Variância Explicada; ^||^Estatisticamente significativo

Considerando apenas a variável Orientação Interpessoal, indicou-se diferenças significativas entre as variáveis, sendo elas: Tempo de Experiência [F(2)= 4,711, *p* = 0,008, R^2^= 0,021], além das interações entre Sexo e Função [F(3)= 3,817, *p* = 0,010, R^2^= 0,026], Serviço e Tempo de Experiência [F(6)= 2,332, *p* = 0,033, R^2^= 0,032], e Sexo, Função e Horas de Trabalho [F(1)= 7,927, *p* = 0,006, R^2^= 0,018].

Ademais, o modelo univariado da variável Conhecimento Profissional apresentou diferenças significativas para Sexo [F(1)= 4,381, *p* = 0,034, R^2^= 0,009], além das interações entre Sexo e Função [F(3)= 3,207, *p* = 0,022, R^2^= 0,021], Serviço e Tempo de Experiência [F(6)= 2,104, *p* = 0,049, *p* = 0,028], bem como Horas de Trabalho e Tempo de Experiência [F(2)= 8,028, *p* < 0,001, R^2^= 0,035]. 

Posteriormente, o modelo univariado, considerando o Conflito Moral, apresentou diferenças significativas para os grupos de Tempo de Experiência [F(2)= 4,350, *p* = 0,013, R^2^= 0,020], além da interação entre Sexo, Função e Horas de Trabalho [F(2)= 13,567, *p* < 0,001, R^2^= 0,032]. 

Por fim, o modelo univariado, levando em conta a variável Significado Moral, apontou diferenças estatisticamente significativas entre os grupos de Tempos de Experiência [F(2)= 4,749, *p* = 0,008, R^2^= 0,021], além da interação entre Sexo, Serviço, Função e Tempo de Experiência [F(2)= 3,190, *p* = 0,041, R^2^= 0,014]. 

A partir disso, realizou-se comparações pareadas entre os grupos. A Tabela 4 apresenta as comparações pareadas por dimensão, que apresentaram diferenças significativas entre os grupos. 

**Tabela 4:** Comparações pareadas dos grupos que mantiveram diferenças estatísticas significativas conforme o modelo univariado (n = 422). Salvador, BA, Brasil, 2022

Dimensão orientação interpessoal					
	Pares	F*	R^2†^	*p* ^*‡*^	I-J^§^
Tempo de experiência	Até 5 anos - 16 anos ou mais	6,586	0,026	0,036^||^	0,364
	6 a 15 anos - 16 anos ou mais	8,619	0,035	0,021^||^	0,400
**Dimensão conhecimento profissional**					
	Pares	F*	R^2†^	*p* ^*‡*^	I-J^§^
Sexo	Feminino-Masculino	4,117	0,010	0,048^||^	-0,308
**Dimensão conflito moral**					
	Pares	F*	R^2†^	*p* ^*‡*^	I-J^§^
Tempo de experiência	16 anos ou mais - até 5 anos	7,719	0,030	0,027^||^	0,522
	16 anos ou mais - 5 a 16 anos	7,695	0,031	0,024^||^	0,493
**Dimensão significado moral**					
	Pares	F*	R^2†^	*p* ^*‡*^	I-J^§^
Tempo de experiência	16 anos ou mais - Até 5 anos	8,017	0,032	0,024^||^	0,507

*F = Teste PERMANOVA; ^†^R^2 =^ Variância Explicada; ^‡^p = Nível de Significância; ^§^I-J = Diferença entre a média da resposta entre os grupos; ^||^Estatisticamente significativo

Os resultados apontam que pessoas com 16 anos ou mais de experiência apresentaram níveis de Orientação Interpessoal superiores, quando comparados a pessoas com tempo de experiência de até 5 anos (I-J= 0,364, *p* = 0,021), e de 6 a 15 anos (I-J= 0,400, *p* = 0,021).

As comparações pareadas indicaram que mulheres apresentaram menores níveis de Conhecimento Profissional, quando comparadas a homens (I-J= -0,308, *p* = 0,048). 

As profissionais com 16 anos ou mais de experiência apresentaram níveis de Conflito Moral superiores, quando comparados com as profissionais com tempo de experiência de até 5 anos (I-J= 0,522, *p* = 0,027) e profissionais com tempo de experiência de 5 a 16 anos (I-J= 0,493, *p* = 0,024). 

As enfermeiras com tempo de experiência de 16 anos ou mais apresentaram maiores níveis de Significado Moral, quando comparados a pessoas com experiência de até 5 anos (I-J= 0,507, *p* = 0,024).

Nas demais variáveis que se apresentaram no modelo univariado por dimensão, não foi possível observar nenhuma outra comparação pareada, levando a crer que os efeitos globais encontrados para as interações não se confirmaram quando comparados os grupos par a par.

## Discussão

Da análise da sensibilidade moral das enfermeiras que atuam nos serviços de urgência no Brasil, alguns destaques foram dados às suas características pessoais e de trabalho. De início, ao descrever as participantes, percebe-se que a distribuição da amostra por sexo apresentou-se majoritariamente feminina, semelhante a outros contextos em que foi estudada a SM em profissionais de enfermagem, como nas Unidades de Terapia Intensiva (UTI) da Turquia^7^, em cuidados de fim de vida na Coreia do Sul^15^ e entre enfermeiros brasileiros^16^. 

Difere essa proporção em relação a outro estudo no contexto da urgência na Turquia, em que foi apresentada uma amostra mais homogênea entre os sexos, revelando 52,5% de mulheres e 47,5% de homens^17^. Já em outro estudo conduzido em clínicas médicas no Irã, a proporção de mulheres alcançou 96,5%^18^. Isso pode indicar que, embora o perfil dos profissionais venha se alterando ao longo dos anos, a Enfermagem ainda se constitui em uma profissão com uma expressiva participação de mulheres, em função da historicidade e questões sociais relacionadas à constituição da profissão. 

A respeito da divergência quanto à influência do sexo do profissional na sua SM, entende-se que avaliar de forma isolada essa característica pode suscitar interpretações equivocadas. Em um estudo que traz diferenças da sensibilidade moral quanto ao sexo dos profissionais, não houve diferenças significativas entre homens e mulheres^7^. No entanto, os dados apresentados nesta pesquisa indicam diferença significativa em relação à dimensão conhecimento profissional, que retratam a confiança no conhecimento da enfermagem e a valorização do conhecimento intuitivo e da experiência pelos profissionais para auxiliá-las a tomar decisões difíceis, sendo que os homens apresentaram maiores índices.

Quando se trata do conhecimento profissional, evidenciou-se em estudo com enfermeiras de UTI na Região Sul do Brasil que a busca pelo conhecimento promove aptidão para questionar os fatos que ocorrem e reconhecer situações inadequadas no contexto de trabalho em que se está inserido^6^, sendo assim, a dimensão se apresenta como importante no alcance do desenvolvimento da sensibilidade moral das profissionais. 

Cabe salientar que a SM não se desenvolve da mesma forma em todos os aspectos da vida^19^, ou seja, uma pessoa pode ter maior sensibilidade em determinados contextos do que em outros, o que eleva a importância da experiência para o desenvolvimento da sensibilidade moral no âmbito da atuação profissional. A experiência profissional, neste estudo, foi considerada quanto ao tempo de atuação no serviço de urgência. 

No cognitivismo, existe a acepção que os princípios morais são frutos do conhecimento inato ou adquirido, de tipo intuitivo ou demonstrativo^2^. Assim, a qualidade das experiências e a cognição (conhecimentos e habilidades apreendidas) desenvolvem as percepções de como as ações influenciam os outros, sendo que as memórias e julgamentos estão envolvidos no processo. 

O clima ético interfere no desenvolvimento das competências morais do indivíduo, assim, os ambientes onde não são valorizadas as questões éticas, a identificação dos problemas e a sua resolução podem gerar prejuízos à sensibilidade moral, levando a um fenômeno que é reconhecido como neutralização moral^3^.

Isto é relevante porque uma das características da sensibilidade é a utilização das experiências profissionais como ferramenta para reconhecer os aspectos éticos envolvidos em determinada situação. Por isso, em melhores condições do desenvolvimento moral, quanto maior o tempo de prática, maior tende a ser a competência moral das enfermeiras para usar habilidades desenvolvidas ao longo do tempo na tomada de decisões^20^. 

Outrossim, os achados deste estudo corroboram o tempo de experiência na urgência como a característica profissional que se destacou nas distinções entre os grupos nas dimensões da SM. Dessa maneira, ao se tratar da dimensão Orientação Interpessoal, os achados sugerem que pessoas com 16 anos de experiência ou mais apresentam níveis superiores na orientação quando comparados a pessoas com tempo de experiência de até 5 anos. 

A dimensão da orientação interpessoal foi a que apresentou maiores concordâncias entre enfermeiras que atuavam em distintos contextos, incluindo também estudos com populações culturalmente distintas^4^. 

Tal dimensão corresponde aos comportamentos desenvolvidos pelos profissionais em busca de uma relação de confiança com o paciente e de alternativas para atender às necessidades^13^, pois existe uma preocupação profissional de como as suas ações afetam a sua relação com a pessoa sob cuidado^14^. Em estudo com equipe de saúde da Atenção Primária à Saúde (APS), a relação interpessoal foi identificada pelos participantes como promotora de novas percepções, que favorecem o desenvolvimento da SM^4^. 

As relações de confiança entre a enfermeira e pessoas sob cuidados quando não são estabelecidas, reforçam o surgimento de problemas éticos^21^. Fortalecer essas relações não se limita ao âmbito do saber fazer um aspecto mais técnico da assistência, mas também integra dimensões da esfera humana, as quais são interconectadas com o respeito, a partir do reconhecimento pelas enfermeiras da singularidade da pessoa sob cuidado e da demonstração de que, para exercer a profissão, o respeito à condição humana é fundamental, assim como as relações multiprofissionais devem ser estabelecidas em prol do provimento do melhor cuidado^5^. 

Nesse sentido, quando enfermeiras buscam conhecer o paciente em sua totalidade, elas têm melhores condições de estabelecer uma relação respeitosa como as percebidas em serviço de urgência^21^. Em estudo sobre a relação da sensibilidade moral e o clima ético do trabalho, foi percebida uma associação positiva da sensibilidade para a manutenção da privacidade da pessoa sob cuidado^22^. Também, elas passam a reconhecer as necessidades das pessoas sob cuidados, por vezes veladas pela dinâmica do serviço, assim, a sensibilidade moral proporciona habilidades para reconhecer e enfrentar os conflitos^5^.

No âmbito da dimensão Conflito Moral, as enfermeiras com mais tempo de experiência são aquelas que mais percebem a vivência do conflito. A SM é um marco da competência moral, por estar envolvida tanto no reconhecimento quanto na melhoria das habilidades para a resolução do conflito^5^. É pela consciência moral que a enfermeira recruta seus conhecimentos sobre a ética profissional e se torna ciente dos possíveis conflitos no seu ambiente de trabalho^20^. Assim, a experiência, quando a vivência é reflexionada, baseia a resolução de conflitos semelhantes no futuro. 

No entanto, os enfermeiros, por vezes, deparam-se com situações em que os valores profissionais divergem dos valores institucionais, ou também sentem dificuldade para lidar com outros colegas e profissionais de trabalho quando estão diante de um paciente^23^. 

Essas situações reforçam a compreensão da necessidade do significado moral da prática, o qual não deve ser estruturado de forma isolada conforme as expectativas externas do atendimento, principalmente quando estes divergem do bem maior, que é a produção do cuidado. Afinal, o cuidado que a enfermagem propõe, como meio para alcançar a finalidade do bem-estar do outro, pauta-se em princípios que garantam o respeito da pessoa sob cuidado mesmo diante dos limites da instituição^24^, por exemplo.

Em relação ao Significado moral das enfermeiras, foi evidenciada a relação existente entre esta dimensão e o tempo de experiência: refere-se a dar sentido às ações tomadas para atender às necessidades da pessoa sob cuidado^14^. Assim, infere-se que os contextos que apresentam redução da autonomia da pessoa, como o que se observa em serviços de urgência^25^, podem levar as enfermeiras a se sentirem moralmente mais responsáveis pela assistência prestada. Em função disso, parece haver maior concordância no significado moral diante das situações em que vivenciam conflitos, como aquelas nas quais é preciso tomar decisões sem a participação da pessoa.

As limitações deste estudo surgem a partir do não estabelecimento de uma amostra probabilística, o que incide na restrição para generalização dos dados. Também, pela necessidade de obter avaliações para estabelecer pontos de corte do instrumento; além da baixa adesão das participantes, que pode estar relacionada à restrição de acesso às redes de internet em algumas regiões.

Entre as contribuições do estudo, destaca-se que se pode favorecer uma melhor compreensão sobre a SM de enfermeiras que atuam em serviços de urgência no contexto brasileiro, a partir da ampliação da temática neste campo de atuação. Tal compreensão é relevante, pois aponta para a necessidade de haver, desde a formação profissional, a promoção de práticas e experiências significativas, que contribuam para o desenvolvimento da SM das futuras enfermeiras e, em consequência, fortaleçam suas decisões com base no cuidado ético e centrado no paciente.

## Conclusão

Ao analisar a sensibilidade moral das enfermeiras da urgência integrando com dados pessoais e de trabalho, na amostra estudada, foi possível identificar que o tempo de experiência no serviço incide em maiores índices do fenômeno em estudo. Ademais, o sexo masculino representou maiores índices de confiança no conhecimento, o que convida a aprofundar as questões de gênero e confiança profissional. 

Destaca-se que valorizar o compartilhamento de vivências e experiências intergeracionais em serviço com as profissionais pode ser uma possível estratégia para fomentar competências morais no campo de prática, ao mesmo tempo que valoriza o corpo de conhecimento constituído ao longo do tempo. No entanto, devido às limitações metodológicas do estudo, em destaque para o tipo de amostragem, sugere-se parcimônia quanto às generalizações dos dados.

# Translation: Sensibilidad moral como atributo personal y laboral de las enfermeras en urgencias: estudio transversal*

**DOI:** 10.1590/1518-8345.7178.4310

**Authors:** Mariana Oliveira Antunes Ferraz, Carlise Rigon Dalla Nora, Maria Manuela Ferreira Pereira da Silva Martins, Rosinete Souza Barata, Larissa Dantas Ferreira, Darci de Oliveira Santa Rosa

**Affiliations:** 1 Universidade Estadual do Sudoeste da Bahia, Jequié, BA, Brasil.; 2 Universidade Federal do Rio Grande do Sul, Escola de Enfermagem, Porto Alegre, RS, Brasil.; 3 Universidade do Porto Instituto de Ciências Biomédicas Abel Salazar, Ciências Biomédicas, Porto, PO, Portugal.; 4 Universidade Estadual de Montes Claros, Enfermagem, Montes Claros, MG, Brasil.; 5 Universidade Federal da Bahia, Escola de Enfermagem, Salvador, BA, Brasil.

**Keywords:** Atención en Enfermería, Urgencias Medicas, Enfermería de Urgencia, Enfermeras y Enfermeros, Ética en Enfermería, Principios Morales

## Abstract

**Objetivo:**

analizar la sensibilidad moral de las enfermeras que trabajan en servicios brasileños de urgencia según sus características personales y laborales.

**Método:**

investigación cuantitativa, descriptiva, transversal y con muestra por conveniencia. Participaron 422 enfermeras de los servicios de atención a la urgencia de las cinco regiones del territorio brasileño. Se recopilaron información sociodemográfica y laboral, y la versión brasileña del *Moral Sensitivity Questionnaire*. Los datos fueron recogidos, tras la aprobación en el Comité de Ética, mediante un formulario autoaplicado en la Plataforma *Google Forms* y sometidos a organización por el *software Excel* y analizados vía lenguaje R.

**Resultados:**

las enfermeras con mayor tiempo de experiencias en los servicios de atención a las urgencias mostraron mayores niveles en las dimensiones orientación interpersonal, conflicto moral y significado moral; en la dimensión conocimiento profesional, los hombres mostraron mayores niveles, evidenciado por ítems que incluyen la confianza en el conocimiento de enfermería, en la intuición, experiencia y opinión.

**Conclusión:**

las distinciones de la sensibilidad moral de las enfermeras se dieron en cuanto a la experiencia profesional. Se destaca que valorar el compartir vivencias y experiencias intergeneracionales en servicio, puede ser una posible estrategia para fomentar competencias morales en el campo de práctica.

## Destacados

**(1)** Comprensión sobre la sensibilidad moral (SM) de enfermeras.

**(2)** Sensibilidad moral de las enfermeras de urgencia.

**(3)** Valorar el compartir vivencias y experiencias intergeneracionales.

**(4)** Estrategia para fomentar competencias morales.

**(5)** Distinciones de la sensibilidad moral relacionada con la experiencia profesional.

## Introducción

Hay situaciones que pueden interferir en el desempeño del cuidado seguro y calificado de las enfermeras en el contexto de los servicios de atención a las urgencias, como por ejemplo, los procesos de trabajo y las relaciones interpersonales establecidas en este escenario de cuidado^1^. Estas situaciones pueden hacer evidentes, o no, posibles conflictos experimentados por las enfermeras, en lo que concierne a sus deberes, responsabilidades y valores durante la prestación de cuidados en salud. De este modo, tomar decisiones frente a la percepción de los conflictos morales, aquí entendidos como todo acontecimiento en el cual se viva contradicción, oposición o enfrentamiento acerca de principios, valores, actitudes^2^, requiere de estas profesionales una serie de competencias.

Entre las competencias evocadas para reconocer situaciones sensibles desde el punto de vista ético y resolver los conflictos, la competencia moral se vuelve indispensable para la práctica asistencial en salud. Esto es porque la calidad del cuidado está tanto relacionada con las habilidades clínicas como con las morales de las enfermeras^3^.

El componente de la competencia moral involucrado en el inicio del proceso reflexivo y de reconocimiento de las situaciones moralmente sensibles es la sensibilidad moral (SM), relevante para que el profesional pueda manejar frente a las complejidades del cuidado, especialmente en contextos de mayores vulnerabilidades^4^. Esta sensibilidad funciona como un despertar individual de las cuestiones morales involucradas en un contexto, que hace que las fuentes de conflictos sean percibidas y enfrentadas. Así, reconocer los conflictos derivados de la práctica del cuidado se convierte en elemento clave disparador del proceso de toma de decisiones y sin esto puede haber compromiso en la calidad de la asistencia de enfermería^5^.

Como la sensibilidad moral es un fenómeno complejo presente en contextos de formación y práctica profesional, y diversos elementos interactúan en su desarrollo, es que se fundamenta el profundizar del fenómeno con atención a los distintos escenarios de actuación de la enfermería^4^.

El ejercicio profesional favorece el desarrollo de la sensibilidad moral, pues es en este contexto donde se percibe la real dimensión de los problemas éticos^6^. Así, la experiencia de las enfermeras ha sido asociada al desarrollo de la sensibilidad moral^7^ ^)-(^ ^8^. Algunos estudios buscaron establecer relaciones entre características individuales y laborales, como sexo y lugar de trabajo, con la sensibilidad moral, con distintas conclusiones acerca de estas asociaciones^7^ ^),(^ ^9^ ^)-(^ ^10^.

En revisión de la literatura sobre la interacción entre sensibilidad moral y el cuidado, tres estudios abordaron la sensibilidad moral de las enfermeras en la atención primaria a la salud y unidades hospitalarias en Brasil^4^. No hay estudios que profundicen la sensibilidad moral en el contexto de las urgencias brasileñas. Sin embargo, estudios internacionales fueron realizados con los profesionales de enfermería en el contexto de los servicios de urgencias, como en Irán, donde se promovió un programa de empoderamiento para los enfermeros y en la evaluación, el impacto fue una mayor sensibilidad moral en el grupo de la intervención^11^.

En la búsqueda por profundizar los conocimientos del fenómeno en el contexto de las urgencias, dada las especificidades del cuidado en esta área, el estudio tiene como objetivo analizar la sensibilidad moral de las enfermeras que actúan en servicios brasileños de urgencia conforme sus características personales y laborales.

## Método

### Tipo del estudio

Se trata de una investigación cuantitativa y transversal, cuyo informe fue orientado por los ítems de verificación *Strengthening the Reporting of Observational Studies in Epidemiology* (STROBE) para estudios observacionales.

### Período

La recolección de datos ocurrió en el período de febrero a junio de 2022.

### Población

En la investigación nacional de enfermería, existen 85.773 enfermeras y enfermeros activos en la Asistencia Hospitalaria en Urgencia y Emergencia, UPA y SAMU, en todo Brasil^12^. Las participantes del estudio fueron enfermeras activas en servicios brasileños de urgencia.

### Criterios de selección

El criterio para la inclusión del participante fue la actuación como enfermera en servicios de atención a la urgencia, y fueron excluidas aquellas entre los elegibles que se negaron a registrar concordancia, virtualmente, del Consentimiento Informado (CI), y las que no ejercían la función en el momento de la recolección, presentada en una pregunta de exclusión del proceso de llenado del instrumento.

### Participantes

La muestra fue no probabilística, por conveniencia debido al período de realización del estudio que ocurrió aún en el período pandémico, en que el acceso a los potenciales participantes era restringido.

Participaron del estudio 422 enfermeras activas en servicios brasileños de urgencia, captadas a partir de la espontaneidad de los profesionales en acceder y responder el instrumento. Se realizaron 751 accesos al instrumento en el período de recolección de datos, 11 personas no estuvieron de acuerdo con el CI o no aceptaron participar del estudio, 319 no trabajaban en servicios de urgencia en el momento del llenado, lo que resultó en los participantes de este estudio. 

### Instrumentos utilizados para la recolección de las informaciones

Para evaluar la sensibilidad moral de las enfermeras se insertó el Cuestionario de Sensibilidad Moral (MSQ-B), traducido y validado en Brasil^13^. Para este estudio se consideraron 19 ítems previamente sometidos al estudio de evaluación de la evidencia de validez de estructura interna, en que presentó buenos índices de consistencia interna, evaluada a partir del omega de McDonald ωMSQ-B-19= 0,812.

Los ítems estaban distribuidos en las dimensiones: orientación interpersonal, la motivación del contacto social, percibida en la enfermería como la atención en la relación en busca de la confianza; significado moral, se refiere a dar sentido a las acciones tomadas a partir de la reflexión y estructuración del sentido; vivencia del conflicto moral, se refiere a la percepción sobre el conflicto; y confianza en el conocimiento, retrata la convicción de que el conocimiento es necesario para manejar las cuestiones morales^13^ ^-^ ^14^. Las respuestas se obtenían en una escala tipo Likert de 1 (discrepo totalmente) a 7 (estoy totalmente de acuerdo). 

### Recolección de datos

La recolección de datos se llevó a cabo en formato *online* por medio de un formulario autoaplicado en la Plataforma *Google Forms* que contenía la versión brasileña del *Moral Sensitivity Questionnaire*, cuya divulgación se realizó en redes sociales para alcanzar las cinco regiones brasileñas.

Contribuyeron a la divulgación del instrumento *online*, la red de contactos de las investigadoras, los Consejos Regionales de Enfermería y los Programas de Posgrado en Enfermería, contactados por la investigadora responsable, quien solicitó apoyo en las redes sociales, ocurriendo por correo electrónico y/o Instagram.

### Tratamiento y análisis de los datos

Los datos fueron tabulados en el *software Excel* y analizados mediante el lenguaje de programación R (R CORE TEAM). Se realizó un Análisis Multivariado de Varianza por Permutaciones (PERMANOVA), ya que se trataba de datos asimétricos. El supuesto de normalidad de la muestra fue investigado mediante la prueba de Kolmogorov-Smirnov y los valores considerados significativos en cuanto al p-valor fueron de 0,05.

A partir de esto, el modelo multivariado fue probado utilizando como variables dependientes los factores del cuestionario de sensibilidad moral, siendo ellos: a) Orientación Interpersonal; b) Conocimiento Profesional; c) Conflicto Moral; d) Significado Moral.

Las variables independientes (i.e., delimitadoras de grupos), fueron: I) Sexo (femenino y masculino); II) Servicio (emergencia hospitalaria, Servicio de Atención Móvil de Urgencia 192, Unidades de Pronta Atención y otros servicios de urgencia); III) Función (asistencia directa, coordinación, regulación y otra actividad considerada por el participante); IV) Horas de trabajo (hasta 30 horas, 31 a 44 horas y más de 44 horas); y V) Tiempo de experiencia (hasta 5 años, 6 a 15 años y más de 16 años).

### Aspectos éticos

El estudio obtuvo aprobación por el Comité de Ética en Investigación de la *Escola de Enfermagem* de la *Universidade Federal da Bahia*, bajo el dictamen nº 5.141.402 y Certificado de Presentación de Apreciación Ética (CAAE) 53607021.2.0000.5531. Las participantes fueron informadas y esclarecidas sobre los objetivos, beneficios y riesgos de la investigación y los contactos de las investigadoras para dudas y esclarecimientos adicionales presentes en el CI, cuya copia fue enviada al correo electrónico registrado por cada participante.

## Resultados

De las participantes, 322 (78,7%) fueron del sexo femenino, por lo que se optó por reportar en el estudio el término enfermera(s) para la descripción en el estudio. La mayor parte de la muestra trabajaba en el servicio de emergencia hospitalaria en el momento de la recolección de datos (n= 145, 34,4%), la función de asistencia directa al paciente presentó la mayor parte de las participantes (n= 329, 78%), además del tiempo de experiencia en el servicio de urgencia de hasta 5 años (n= 181, 42,9%), y las horas de trabajo en el rango de más de 30 hasta 44 horas (n= 253, 60%). A continuación se presentan las demás características de las participantes así como las pruebas realizadas (Tabla 1).

**Tabla 1:** Perfil de las enfermeras activas en los servicios de urgencia según características personales y profesionales (n = 422). Salvador, BA, Brasil, 2022

Variables		n*	%
**Sexo**	Femenino	332	78,67%
	Masculino	90	21,33%
**Servicio**	Emergencia hospitalaria	145	34,36%
	Otro servicio de Urgencia y Emergencia	92	21,80%
	SAMU^†^ 192	62	14,69%
	UPA^‡^ 24 horas / Atención inmediata	123	29,15%
**Función**	Atención directa al paciente	329	77,96%
	Coordinación	70	16,59%
	Otra actividade	18	4,26%
	Regulación	5	1,18%
**Horas de trabajo**	Hasta 30 horas	106	25,12%
	De 31 a 44 horas	253	59,95%
	Más de 44 horas	63	14,93%
**Tiempo de experiencia**	Hasta 5 años	181	42,89%
	De 6 a 15 años	177	41,94%
	16 años o más	64	15,17%

*n = Frecuencia Absoluta; ^†^SAMU = Servicio de Atención Móvil de Urgencia; ^‡^UPA = Unidad de Pronta Atención

Inicialmente, las pruebas de normalidad fueron investigadas mediante el test de Kolmogorov-Smirnov, que señalaron la asimetría en la distribución de las variables dependientes en todos los casos. De esta manera, las pruebas inferenciales que se siguen están configuradas en alternativas no paramétricas para la investigación.

A partir de esto, el modelo multivariado se realizó considerando 9.999 remuestreos. Los resultados de la PERMANOVA indicaron diferencias estadísticamente significativas solo para las variables Tiempo de Experiencia [F(2)= 3,540, *p* = 0,032, R^2= 0,016], además de las interacciones entre Horas de Trabajo y Tiempo de Experiencia [F(2)= 3,126, *p* = 0,045, R^2= 0,014], y entre Sexo, Función y Horas de Trabajo [F(1)= 10,518, *p* = 0,002, R^2= 0,024] (Tabla 2).

**Tabla 2:** Modelo multivariado de la sensibilidad moral (n = 422). Salvador, BA, Brasil, 2022

	F*	df^†^	*p* ^*‡*^	R^2§^
Sexo	2,471	1	0,115	0,005
Servicio	1,168	3	0,318	0,008
Función	0,221	3	0,882	0,001
Horas de trabajo	1,285	1	0,260	0,003
Tiempo de experiencia	3,540	2	0,032^||^	0,016
Horas de trabajo/Tiempo de experiencia	3,126	2	0,045^||^	0,014
Sexo/Función/Horas de trabajo	10,518	1	0,002^||^	0,024

*F = Prueba PERMANOVA; ^†^df = Grados de Libertad; ^‡^p = Nivel de Significancia; ^§^R^2^ = Varianza Explicada; ^||^Estadísticamente significativo

La Tabla 3 presenta resultados sobre los modelos univariados de los factores/dimensiones del instrumento.

**Tabla 3:** Modelos univariados por dimensión de la sensibilidad moral (n = 422). Salvador, BA, Brasil, 2022

Variables	F*	df^†^	*p* ^*‡*^	R^2§^
**Dimensión Orientación Interpersonal**				
Sexo	0,883	1	0,352	0,002
Servicio	0,645	3	0,593	0,004
Función	0,612	3	0,613	0,004
Horas de trabajo	2,869	1	0,089	0,006
Tiempo de experiencia	4,711	2	0,008^||^	0,021
Sexo/Función	3,817	3	0,010^||^	0,026
Servicio/Tiempo de experiencia	2,332	6	0,033^||^	0,032
Sexo/Función/Horas de trabajo	7,927	1	0,006^||^	0,018
**Dimensión Conocimiento Profesional**				
Sexo	4,381	1	0,034^||^	0,009
Servicio	0,943	3	0,415	0,006
Función	0,775	3	0,520	0,005
Horas de trabajo	0,177	1	0,672	0,000
Sexo/Función	3,207	3	0,022^||^	0,021
Servicio/Tiempo de experiencia	2,104	6	0,049^||^	0,028
Horas de trabajo/Tiempo de experiencia	8,028	2	< 0,001^||^	0,035
**Dimensión Conflicto Moral**				
Sexo	0,939	1	0,331	0,002
Servicio	1,433	3	0,230	0,010
Función	0,189	3	0,903	0,001
Horas de trabajo	0,182	1	0,669	0,000
Tiempo de experiencia	4,350	2	0,013^||^	0,020
Sexo/Función/Horas de trabajo	13,567	2	< 0,001^||^	0,032
**Dimensión Significado Moral**				
Sexo	0,243	1	0,622	0,000
Servicio	2,340	3	0,076	0,015
Función	1,160	3	0,329	0,007
Horas de trabajo	0,331	1	0,557	0,000
Tiempo de experiencia	4,749	2	0,008^||^	0,021
Sexo/Servicio/Función/Tiempo de experiencia	3,190	2	0,041^||^	0,014

*F = Prueba PERMANOVA; ^†^df = Grados de Libertad; ^‡^p = Nivel de Significancia; ^§^R^2^ = Varianza Explicada; ^||^Estadísticamente significativo

Considerando solo la variable Orientación Interpessoal, se indicaron diferencias significativas entre las variables, siendo ellas: Tiempo de Experiencia [F(2)= 4,711, *p* = 0,008, R^2= 0,021], además de las interacciones entre Sexo y Función [F(3)= 3,817, *p* = 0,010, R^2= 0,026], Servicio y Tiempo de Experiencia [F(6)= 2,332, *p* = 0,033, R^2= 0,032], y Sexo, Función y Horas de Trabajo [F(1)= 7,927, *p* = 0,006, R^2= 0,018].

Además, el modelo univariado de la variable Conocimiento Profesional presentó diferencias significativas para Sexo [F(1)= 4,381, *p* = 0,034, R^2= 0,009], además de las interacciones entre Sexo y Función [F(3)= 3,207, *p* = 0,022, R^2= 0,021], Servicio y Tiempo de Experiencia [F(6)= 2,104, *p* = 0,049, R^2= 0,028], además de Horas de Trabajo con Tiempo de Experiencia [F(2)= 8,028, *p* < 0,001, R^2= 0,035].

Posteriormente, el modelo univariado considerando el Conflicto Moral presentó diferencias significativas para los grupos de Tiempo de Experiencia [F(2)= 4,350, *p* = 0,013, R^2= 0,020], además de la interacción entre Sexo, Función y Horas de Trabajo [F(2)= 13,567, *p* < 0,001, R^2= 0,032].

Por último, el modelo univariado considerando la variable Significado Moral señaló diferencias estadísticamente significativas entre los grupos de Tiempos de Experiencia [F(2)= 4,749, *p* = 0,008, R^2= 0,021], además de la interacción entre Sexo, Servicio, Función y Tiempo de Experiencia [F(2)= 3,190, *p* = 0,041, R^2= 0,014].

A partir de esto, se realizaron comparaciones pareadas entre los grupos. La Tabla 4 presenta las comparaciones pareadas por dimensión que presentaron diferencias significativas entre los grupos.

**Tabla 4:** Comparaciones pareadas de los grupos que mantuvieron diferencias estadísticas significativas según el modelo univariado (n = 422). Salvador, BA, Brasil, 2022

Dimensión orientación interpersonal					
	Pares	F*	R^2†^	*p* ^*‡*^	I-J^§^
Tiempo de experiencia	Hasta 5 años - 16 años o más	6,586	0,026	0,036^||^	0,364
	6 a 15 años - 16 años o más	8,619	0,035	0,021^||^	0,400
**Dimensión conocimiento profesional**					
	Pares	F*	R^2†^	*p* ^*‡*^	I-J^§^
Sexo	Femenino-Masculino	4,117	0,010	0,048^||^	-0,308
**Dimensión conflicto moral**					
	Pares	F*	R^2†^	*p* ^*‡*^	I-J^§^
Tiempo de experiencia	16 años o más - hasta 5 años	7,719	0,030	0,027^||^	0,522
	16 años o más - 5 a 16 años	7,695	0,031	0,024^||^	0,493
**Dimensión significado moral**					
	Pares	F*	R^2†^	*p* ^*‡*^	I-J^§^
Tiempo de experiencia	16 años o más - hasta 5 años	8,017	0,032	0,024^||^	0,507

*F = Prueba PERMANOVA; ^†^R^2 =^ Varianza Explicada; ^‡^p = Nivel de Significancia; ^§^I-J = Diferencia entre el promedio de la respuesta entre los grupos; ^||^Estadísticamente significativo

Los resultados indican que las personas con 16 años o más de experiencia presentaron niveles de Orientación Interpersonal superiores, cuando se comparan con personas con tiempo de experiencia de hasta 5 años (I-J= 0,364, *p* = 0,021), y de 6 a 15 años (I-J= 0,400, *p* = 0,021).

Las comparaciones pareadas indicaron que las mujeres presentaron menores niveles de Conocimiento Profesional cuando se comparan con los hombres (I-J= -0,308, *p* = 0,048).

Las profesionales con 16 o más años de experiencia presentaron niveles de Conflicto Moral superiores, cuando se comparan con las profesionales con tiempo de experiencia de hasta 5 años (I-J= 0,522, *p* = 0,027), y profesionales con tiempo de experiencia de 5 a 16 años (I-J= 0,493, *p* = 0,024).

Las enfermeras con tiempo de experiencia de 16 años o más presentaron mayores niveles de Significado Moral cuando se comparan con personas con experiencia de hasta 5 años (I-J= 0,507, *p* = 0,024).

En las demás variables que se presentaron en el modelo univariado por dimensión, no fue posible observar ninguna otra comparación pareada, lo que lleva a creer que los efectos globales encontrados para las interacciones no se confirmaron cuando se compararon los grupos de manera pareada.

## Discusión

Desde el análisis de la sensibilidad moral de las enfermeras que actúan en los servicios de urgencia en Brasil, se destacaron ciertas características personales y laborales. Al principio, al describir a las participantes, se percibe que la distribución de la muestra por sexo fue predominantemente femenina, similar a otros contextos en los que se estudió la SM en profesionales de enfermería, como en las Unidades de Cuidados Intensivos (UCI) de Turquía^7^, en cuidados de fin de vida en Corea del Sur^15^ y entre enfermeros brasileños^16^.

Esta proporción difiere de otro estudio en el contexto de urgencia en Turquía, donde se presentó una muestra más homogénea entre los sexos, revelando 52,5% de mujeres y 47,5% de hombres^17^. En otro estudio realizado en clínicas médicas en Irán, la proporción de mujeres alcanzó el 96,5%^18^. Esto puede indicar que, aunque el perfil de los profesionales ha cambiado a lo largo de los años, la enfermería sigue siendo una profesión con una significativa participación de mujeres, debido a la historicidad y cuestiones sociales relacionadas con la constitución de la profesión.

En cuanto a la existencia de divergencias respecto a la influencia del sexo del profesional en su SM, evaluar de forma aislada esta característica puede suscitar interpretaciones equivocadas. Un estudio que examinó las diferencias de sensibilidad moral según el sexo de los profesionales no encontró diferencias significativas entre hombres y mujeres^7^. Sin embargo, los datos presentados en esta investigación indican una diferencia significativa en relación con la dimensión conocimiento profesional, que refleja la confianza en el conocimiento de enfermería y la valoración del conocimiento intuitivo y de la experiencia por los profesionales para ayudarles a tomar decisiones difíciles, siendo que los hombres presentaron mayores índices.

En lo que se refiere al conocimiento profesional, se demostró en un estudio con enfermeras de UCI en la Región Sur de Brasil que la búsqueda de conocimiento promueve la habilidad para cuestionar los hechos que ocurren y reconocer situaciones inadecuadas en el contexto laboral en el que están insertadas^6^, siendo, por tanto, una dimensión importante para alcanzar el desarrollo de la sensibilidad moral de estas profesionales.

Es importante destacar que la SM no se desarrolla de la misma manera en todos los aspectos de la vida^19^, es decir, una persona puede tener mayor sensibilidad en ciertos contextos que en otros, lo que eleva la importancia de la experiencia para el desarrollo de la sensibilidad moral en el ámbito de la actuación profesional. La experiencia profesional en este estudio se consideró en cuanto al tiempo de actuación en el servicio de urgencia.

En el cognitivismo, existe la concepción de que los principios morales son frutos del conocimiento innato o adquirido, de tipo intuitivo o demostrativo^2^. Así, la calidad de las experiencias y la cognición (conocimientos y habilidades aprendidas) desarrollan las percepciones de cómo las acciones influyen en los demás, estando involucrados en este proceso las memorias y juicios.

El clima ético interfiere en el desarrollo de las competencias morales del individuo, así, los ambientes donde no se valoran las cuestiones éticas, la identificación de los problemas y su resolución, pueden generar perjuicios a la sensibilidad moral, llevando a un fenómeno que se reconoce como neutralización moral^3^.

Esto es relevante porque una de las características de esta sensibilidad es la utilización de las experiencias profesionales como herramienta para reconocer los aspectos éticos involucrados en una determinada situación. Por ello, en mejores condiciones de desarrollo moral, cuanto mayor es el tiempo de práctica, mayor tiende a ser la competencia moral de las enfermeras para usar habilidades desarrolladas a lo largo del tiempo en la toma de decisiones^20^.

Además, los hallazgos de este estudio corroboran el tiempo de experiencia en la urgencia como la característica profesional que se destacó en las distinciones entre los grupos en las dimensiones de la SM. De este modo, al tratar de la dimensión Orientación Interpessoal, estos hallazgos sugieren que las personas con 16 años de experiencia, o más, presentan niveles superiores en esta orientación cuando se comparan con personas con tiempo de experiencia de hasta 5 años. 

La dimensión de la orientación interpersonal fue la que presentó mayores concordancias entre enfermeras que trabajaban en distintos contextos, incluyendo también estudios con poblaciones culturalmente distintas^4^. Esta dimensión corresponde a los comportamientos desarrollados por los profesionales en busca de una relación de confianza con el paciente y de alternativas para atender a las necesidades^13^, ya que existe una preocupación profesional sobre cómo sus acciones afectan su relación con la persona bajo cuidado^14^. En un estudio con el equipo de salud de la Atención Primaria a la Salud (APS), la relación interpersonal fue identificada por los participantes como promotora de nuevas percepciones, que favorecen el desarrollo de la SM^4^.

Las relaciones de confianza entre la enfermera y las personas bajo cuidado, cuando no se establecen, refuerzan la aparición de problemas éticos^21^. Fortalecer esas relaciones no se limita al ámbito del saber hacer, un aspecto más técnico de la asistencia, sino que integra dimensiones de la esfera humana, las cuales están interconectadas con el respeto, a partir del reconocimiento por parte de las enfermeras de la singularidad de la persona bajo cuidado y demostración de que para ejercer la profesión, el respeto a la condición humana es fundamental, como también las relaciones multiprofesionales deben establecerse en pro del provisión del mejor cuidado^5^.

Así, cuando las enfermeras buscan conocer al paciente en su totalidad, tienen mejores condiciones de establecer una relación respetuosa como las percibidas en servicio de urgencia^21^. En un estudio sobre la relación de la sensibilidad moral y el clima ético del trabajo, se percibió una asociación positiva de esta sensibilidad para el mantenimiento de la privacidad de la persona bajo cuidado^22^. Además, ellas pasan a reconocer necesidades de las personas bajo cuidados, a veces veladas por la dinámica del servicio, así, la sensibilidad moral proporciona habilidades para reconocer y enfrentar los conflictos^5^.

En el ámbito de la dimensión Conflicto Moral, las enfermeras con más tiempo de experiencia son aquellas que más perciben la vivencia del conflicto. La SM es un hito de la competencia moral, por estar involucrada tanto en el reconocimiento como en la mejora de las habilidades para la resolución del conflicto^5^. Es por la conciencia moral que la enfermera recluta sus conocimientos sobre la ética profesional y se vuelve consciente de los posibles conflictos en su ambiente de trabajo^20^. Así, la experiencia, cuando la vivencia es reflexionada, fundamenta la resolución de conflictos similares en el futuro.

Sin embargo, los enfermeros, a veces, se enfrentan a situaciones en las que los valores profesionales divergen de los valores institucionales, o también, sienten dificultad para lidiar con otros colegas y profesionales de trabajo cuando están ante un paciente^23^. 

Estas situaciones refuerzan la comprensión de la necesidad del significado moral de la práctica, el cual no debe estructurarse de forma aislada según las expectativas externas de la atención, especialmente cuando estas divergen del bien mayor, que es la producción del cuidado. Al fin y al cabo, el cuidado que la enfermería propone como medio para alcanzar la finalidad del bienestar del otro, se basa en principios que garantizan el respeto de la persona bajo cuidado incluso ante los límites de la institución^24^, por ejemplo.

En la dimensión significado moral de las enfermeras, también se evidenció la relación existente de esta dimensión y el tiempo de experiencia, se refiere a dar sentido a las acciones tomadas para atender a la necesidad de la persona bajo cuidado^14^. Así, se infiere que los contextos que presentan reducción de la autonomía de la persona, como observables en servicios de urgencia^25^, pueden llevar a las enfermeras a sentirse moralmente más responsables por la asistencia prestada. En función de esto, parece haber mayor concordancia en el significado moral ante las situaciones en que vivencian conflictos, como aquellas en las cuales toman decisiones sin la participación de la persona.

Las limitaciones de este estudio surgen a partir del no establecimiento de una muestra probabilística, lo que incide en la restricción para la generalización de los datos. También, por la necesidad de obtener evaluaciones para establecer puntos de corte del instrumento; y la baja adhesión de las participantes que puede estar relacionada con la restricción de acceso a las redes de internet en algunas regiones.

Entre las contribuciones del estudio, se destaca que este puede favorecer una mejor comprensión sobre la SM de enfermeras que actúan en servicios de urgencia en el contexto brasileño, a partir de la ampliación de la temática en este campo de actuación. Esta comprensión es relevante, pues señala la necesidad de que, desde la formación profesional, debe haber promoción de prácticas y experiencias significativas, que contribuyan al desarrollo de la SM de las futuras enfermeras y, en consecuencia, fortalezcan sus decisiones basadas en el cuidado ético y centrado en el paciente.

## Conclusión

Al analizar la sensibilidad moral de las enfermeras de urgencia integrando datos personales y de trabajo, en la muestra estudiada, fue posible identificar que el tiempo de experiencia en el servicio incide en mayores índices del fenómeno en estudio. Además, el sexo masculino representó mayores índices de confianza en el conocimiento, lo que invita a profundizar las cuestiones de género y confianza profesional.

Se destaca que valorar el compartimiento de vivencias y experiencias intergeneracionales en servicio con las profesionales, puede ser una posible estrategia para fomentar competencias morales en el campo de práctica, al mismo tiempo que valora el cuerpo de conocimiento constituido a lo largo del tiempo. Sin embargo, debido a las limitaciones metodológicas del estudio, especialmente por el tipo de muestreo, se sugiere prudencia respecto a las generalizaciones de los datos.
